# Dissociating Slow Responses From Slow Responding

**DOI:** 10.3389/fpsyt.2020.505800

**Published:** 2020-10-02

**Authors:** Gayatri Salunkhe, Bernd Feige, Christopher W. N. Saville, Maria Elena Stefanou, David Linden, Stephan Bender, Andrea Berger, Nikolaos Smyrnis, Monica Biscaldi, Christoph Klein

**Affiliations:** ^1^ Department of Child and Adolescent Psychiatry, Psychotherapy and Psychosomatics, Medical Center, Faculty of Medicine, University of Freiburg, Freiburg, Germany; ^2^ Department of Psychiatry and Psychotherapy, Medical Center, Faculty of Medicine, University of Freiburg, Freiburg, Germany; ^3^ School of Psychology, Bangor University, Bangor, United Kingdom; ^4^ School of Psychology and Clinical Language Sciences, University of Reading, Reading, United Kingdom; ^5^ School for Mental Health and Neuroscience, Maastricht University, Maastricht, Netherlands; ^6^ Department of Child and Adolescent Psychiatry, Medical Faculty, University of Cologne, Cologne, Germany; ^7^ Department of Psychology, Ben-Gurion University of the Negev, Beer Sheva, Israel; ^8^ Department of Psychiatry, National and Kapodistrian University of Athens, Medical School, Eginition Hospital, Athens, Greece

**Keywords:** attention-deficit/hyperactivity disorder (ADHD), intra-subject variability, response speed, ex-Gaussian modeling, principal components analyses

## Abstract

Increased Intra-Subject Variability (ISV) is a candidate endophenotype of ADHD. ISV’s relationship with response speed is highly relevant for ADHD as patients are highly variable but typically no slower than controls. This brief report addresses the relationship between variability and speed by employing dimensional analyses for differentiated performance measures, with a particular focus on the ex-Gaussian measures, across relevant ADHD studies and in young healthy adults (N = 70). For both patients with ADHD and healthy adults, we found that reaction time standard deviation and mean reaction time were strongly correlated, thus failing to dissociate, but ex-Gaussian tau (τ) shared only little variance with Gaussian mu (μ), thus dissociating slow responses (τ) from response speed or—if given—slow responding (μ). Our results highlight the utility of employing the ex-Gaussian measures to disentangle ISV and speed, particularly for ADHD data as patients make more slow responses but are not overall slower than typical controls.

## Introduction

Intra-Subject Variability (ISV) refers to short-term within-person variations in performance. Increased ISV of reaction times (RTs) is among the most robust findings and is a candidate endophenotype for Attention Deficit Hyperactivity Disorder [ADHD; (1)]. With regard to ISV’s relationship with response speed, strong correlations between RT standard deviations (RTSD), and mean reaction time (MRT) in healthy (2) and ADHD-related populations (3) suggest that these RT constructs may reflect the same underlying processes. Yet, in ADHD, larger group effect sizes have been observed for RTSD than MRT [ (4); also see **Table 1**] supporting the proposition that response slowing is secondary to a more fundamental deficit of elevated response variability (17).

**Table 1:** Overview of effect sizes of group differences in performance measures in ADHD studies with ex-Gaussian analyses.

Study	S	Task(s)	N Pat	N Con	Age Pat	Age Con	Omi. errors	Comm.errors	σ	τ	RTSD	Mean RTs	μ
Leth-Steensen et al. (5)	*F*	WCRT	17	18	10.83	11.08	–	–	0.16	**2.56** ^c^	**2.47** ^c^	**1.67** ^c^	0.41
Hervey et al. (6)	*F*	CCPT	65	65	10.7	10.6	**0.48** ^b^	0.17	**0.51** ^b^	**0.95** ^c^	**0.86** ^c^	**0.56** ^b^	**↓0.41** ^a^
Vaurio et al. (7)	*F*	Simple & complex GNG	57	83	10.9	11	–	**0.72** ^c^	**0.37** ^a^	**0.46** ^b^	**0.55** ^b^ #	0.20	0.05
Buzy et al. (8)	*F*	VSAT	24	24	10.2	10.3	**0.80** ^b^	0.32	**0.72** ^a^	**0.93** ^b^	–	–	0.09
Epstein et al. (9)	*d*	CDT, CANT, GNG, SST, NBT	51	47	7.90	8.33	–	0.34, **0.63** ^b^ **, 0.55** ^a^ **, 0.71** ^b^ **, 0.85** ^c^	–	**0.90** ^c^ **, 0.89** ^c^ **, 0.86** ^c^ **, 0.85** ^c^ **, 0.76** ^b^	**0.56** ^a^ **, 0.84** ^c^ **, 1.11** ^c^, 0.48**, 0.67** ^b^	0.19, 0.42, **0.64** ^b^, 0.02, 0.30	–
Feige et al. ^1^ (10)	*F*	NBT	27	26	11.1	11.7	**0.87** ^b^	**1.15** ^c^	0.49	**1.19** ^c^	**1.11** ^c^	<0.28	<0.28
Hwang Gu et al. ^1,2,3^ (11)	*F*	CCPT	195	90	12.46	12.63	**0.60** ^c^	**0.40** ^b^	0.22	**0.80** ^c^	**0.82** ^c^	**0.31** ^a^	**↓0.36** ^b^
Tarantino et al. ^1^ (12)	*F*	CCPT	30	30	11.48	11.30	**1.44** ^c^	0.11	**1.13** ^c^	**1.40** ^c^	**1.21** ^c^	**1.11** ^c^	0.37
Borella et al. (13)	*F*	CST, SRT	24	24	9.50	9.29	–	–	<0.3, <0.3	**0.66** ^a^, 0.53	**0.6** ^a^, **0.67** ^a^	0.5, <0.3	0.56, <0.3
Metin et al. (14)	*F*	GNG	25	29	10.20	10.32	**0.58** ^a^	0.44	**0.64** ^a^	**1.01** ^a^	**0.95** ^a^	**0.76** ^a^	0.37
Lee et al. (15)	*F*	GNG	44	31	11.16	11.23	**0.54** ^a^	**0.64** ^b^	0.07	**0.58** ^a^	**0.63** ^a^	0.27	**↓0.59** ^a^
Lin et al. ^1,2,4,5^ (16)	*d*	CCPT	411	138	11.6	11.9	**0.93**	**0.56**	**0.49**	**0.98**	**0.99** $	**0.57**	−0.35

Original test statistics reflecting group differences were converted to Cohen’s d using https://www.psychometrica.de/effect_size.html. Markings in bold if scores significantly differ; with “↓” if scores for patients with ADHD are significantly lower than controls. ^a^p <.05; ^b^p <.01; ^c^p <.001; -, n.s. but no effect sizes provided.

σ, sigma; τ, tau; μ, mu. S, Original statistic; N Pat, Number of patients; N Con, Number of controls; Age Pat, Mean Age of patients; Age Con, Mean age of controls; Cov., Covariates; Omi. Errors, Omission errors; Comm. errors, Commission errors; RT, reaction time; RTSD, RT Standard Deviation; WCRT, Warned 4-Choice RT task; CCPT, Conners’ Continuous Performance Task; GNG, Go/No Go Task; VSAT, Visual Serial Addition Task; CDT, Choice Discrimination Task; CANT, Child Attentional Network Task; SST, Stop Signal Task; NBT, N-Back Task; CST, Color Stroop Test; SRT, Simple RT task; ^#^coefficient of variance (CV); ^$^RT standard errors. Covariates^1,2,3,4,5^: age, sex, IQ, comorbidity, MPH, respectively.

**Figure 1:**
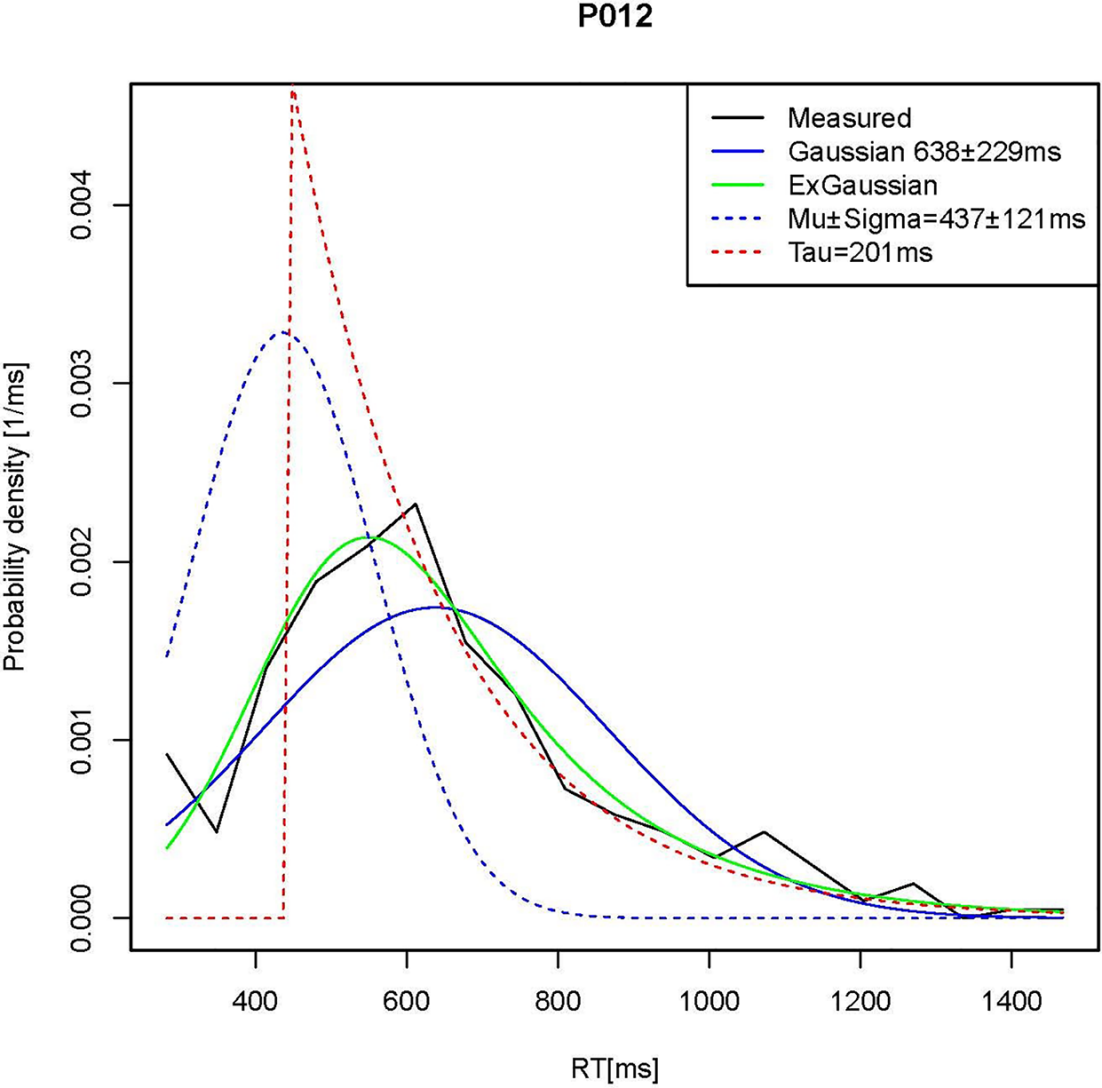
Illustration of the ex-Gaussian analysis. The black line shows the measured RT distribution in one participant the study of Feige et al. (10) that is poorly represented by a Gaussian function (solid blue line). The ex-Gaussian analysis, by contrast, yields a good fit of the empirical distribution (green line) and is composed of the superposition of a Gaussian (hatched blue line) and truncated exponential (hatched red line) function.

By aggregating all deviations from the mean, RTSD reflects a convolution of all kinds of variability [e.g., (non-) linear trends, (non-) periodic fluctuations] and is therefore a sensitive but non-specific indicator of ISV (10). The same is true for MRT in the case of response speed. Besides the lack of specificity of RTSD and MRT, both these measures assume that RT distributions have a Gaussian or normal shape even though this is usually not the case. For both control and ADHD, RT distributions are typically ex-Gaussian with a rightward skew of particularly slow responses that possibly reflect lapses of attention (5). The ex-Gaussian model assumes that RT distributions are a convolution of Gaussian and ex-Gaussian components, and provides the following three parameters to measure these distinct components separately: mu (μ) and sigma (σ) represent the arithmetic mean and standard deviation of the Gaussian portion, respectively, and tau (τ) represents the mean and standard deviation of the ex-Gaussian portion of an RT histogram (**Figure 1**). According to the ex-Gaussian model, the expectancy value of RT equals μ + τ, and the variance of RT equals σ ^2^ + τ^2^ (18).

In patients with ADHD, elevated τ is reported alongside normal or even lower μ (see **Table 1**). This evidence suggesting that patients make a larger number of particularly slow responses but are not generally slower indicates that τ may be dimensionally dissociable from μ. As τ is sensitive to individual differences in ISV (5, 17) its potential dissociation from μ could subsequently disentangle ISV and response speed—two otherwise seemingly overlapping RT constructs (2, 3).

ADHD studies have shown that increased ISV in patients is attributable primarily to τ rather than σ (**Table 1**) and to increased power of RT oscillations at low frequencies (10, 19). These ex-Gaussian distributional and time-series (e.g., frequency-spectral) measures may capture different aspects of or even delineate different underlying processes contributing to ISV (20, 21). A dimensional dissociation of ISV measures from one another would suggest that variability has different “facets”.

In the present brief research report, we are taking a fresh look at the old debate surrounding the close relationship between variability and speed of responding (2, 17) using differentiated measures of ISV (see *Principal Components Analysis for Young Healthy Adults*). We address the dimensional relationship between differentiated RT ISV and speed measures across pertinent ADHD studies (shown in **Table 1**) and for data of young healthy adults. We hypothesize that for ADHD and general populations, ex-Gaussian τ and Gaussian μ dissociate slow responses from slow responding and, consequently, disentangle ISV and speed.

## Methods

### Dimensional Analyses Across Pertinent ADHD Studies

In the summary of 12 studies examining ex-Gaussian measures across a total of 970 ADHD children and 605 healthy controls (**Table 1**), effect sizes (F-values) for differentiated performance measures were converted to Cohen’s *d* using the www.psychometrica.de/effect_size tool. For studies that reported effect sizes of performance measures in more than one task, the Cohen’s *d* values were averaged across tasks to achieve a single value per variable and study. Dimensional analyses between the performance measures were computed using Pearson correlation for pairwise complete observations across the summary of ADHD studies. In a few studies, the effect sizes for specific variables were not investigated or not reported (-), or were reported using a “less than” value (e.g., F < 0.1) rather than a precise value, and were thereby excluded from the analyses. Finally, as bivariate scatterplots revealed that the scores for some variables of the Leth-Steensen et al. study (5) were outliers, correlations excluding this study have been reported additionally as footnotes.

### Principal Components Analysis for Young Healthy Adults

All procedures complied with the Declaration of Helsinki and were approved by the ethics committee at the School of Psychology, Bangor University. Written informed consent was given by all participants before testing. The current work uses the behavioral data from a genotype (COMT Val^158^Met polymorphism) and ERP study (22) and includes data of 70 healthy young Caucasian adults (age 21.1 ± 2.6, 55.7% females, 90% right-handed), mainly students at Bangor University. An *n*-back task with three levels (0-back/1-back/2-back) was administered in two runs. Participants responded to series of letters presented for 1000ms with average stimulus onset intervals of 2s on average, with targets occurring in 25% of the total trials [see Saville et al. for details; (22)].

RT parameters were computed after excluding RTs faster than 200 ms or anticipations. Next, RTs were “residualized” by subtracting the fit to linear models for significant effects of time-on-task (e.g., fatigue, practice effects, and sustained attention), and task/stimuli-related effects such as working memory load (0-back/1-back/2-back), trial-type (targets/non-targets) and run (first/second). This step was conducted to “extract” endogenous variability, or Type I (except time-on-task effects) and II ISV (23). The “residualized” RTs were used to compute the following ISV measures: RTSD, consecutive standard deviation (CSD, assessing trial-to-trial variability using the formula sqrt(∑(RT_i_ − RT_i+1_)^2^/(n − 1)); i = trial number; n = number of trials), σ, τ, and spectral power. Variability was studied in the frequency domain through the computation of spectral power by resampling the residualized RT time series to a frequency of 1 Hz through linear interpolation and by Fast Fourier Transformations (FFT) separately for each participant and each block. FFT scores, derived in bins of 0.004 Hz across the range of 0–0.25Hz, were pooled across bins 0–0.1Hz given as this spectral range is sensitive to increases in ISV in ADHD (10). MRT and μ, computed using non-residualized RTs, were derived as measures of response speed, and percentages of omission errors (no response button pressed) and incorrect responses, as measures of accuracy.

After averaging scores across by task block and removing the COMT genotype-related variances through *z*-transformation, a Principal Component Analysis (PCA) with varimax rotation was run on the following 9 variables: RTSD, CSD, FFT < 0.1 Hz, τ, σ, μ, MRT, omission errors and incorrect responses. The PCA fulfilled assumptions of sampling adequacy (Kaiser-Meyer-Olkin test: KMO = .753) and sphericity (Bartlett’s test: χ²_(36)_ = 1,013.02, *p* <.001). A scree plot confirmed that three factors, with eigenvalues greater than 1, were retainable.

## Results

With regard to dimensional analyses across the pertinent ADHD studies in **Table 1**, a strong association was found between the classical measures of ISV and response speed, RTSD and MRT (*r* = .94^1^). The correlations of RTSD (τ: *r*= .99^2^; σ: *r* = −.06^3^) and MRT (τ: *r* = .96^4^; σ: *r* = .25^5^) with the ex-Gaussian ISV measures are in line with their algebraic dependency. It is striking however that τ, which shared a near-perfect association with RTSD, shared little variance with μ (*r* = .18^6^) across studies.

For young healthy adults, the PCA revealed that ISV, speed and accuracy split into three separate components (see **Table 2**). The exception to this pattern was σ, which loaded on the same factor as speed variables. The three factors explained 90% of inter-individual variance.

**Table 2:** PCA for measures of ISV, speed, and accuracy in young healthy adults.

	Factor 1	Factor 2	Factor 3
τ	**.98**	.08	.07
CSD	**.90**	.30	.22
RTSD	**.89**	.37	.23
FB **≤**.1Hz	**.83**	.41	.30
μ	.19	**.94**	−.12
Mean RTs	.52	**.82**	−.04
σ	.22	**.78**	.39
%incorrect responses	.10	−.11	**.90**
%omission errors	.26	.19	**.76**
*Eigenvalues*	*3.68*	*2.61*	*1.76*
*Proportion of Variance*	*41%*	*29%*	*20%*

σ, sigma; τ, tau; μ, mu; RTSD, reaction time standard deviation; CSD, consecutive standard deviation; FB, frequency bands. Loadings above.75 marked in bold.

Pearson’s correlations suggest that dimensional patterns in healthy adults are overall consistent with those found across ADHD studies. A positive association was present between RTSD and MRT (*r* = .74). Strong to moderate associations of RTSD (τ: *r* = .90; σ: *r* = .62) and MRT (τ: *r* = .60; σ: *r* = .65) with the ex-Gaussian measures support the algebraic dependency of these measures. Importantly, τ and μ shared a comparatively weaker association (*r* = .28) and even loaded highly on separate components of ISV and response speed, respectively. As a control, the exclusion of the ex-Gaussian measures from the PCA resulted in ISV and response speed measures loading highly on a single component.

## Discussion

To begin with, strong associations were found between RTSD and MRT, both across pertinent ADHD studies (**Table 1**) and in young healthy adults. Thus, the close relationship between the conventional measures of ISV and speed, as previously found in healthy adults (2, 17), also seems to exist across ADHD studies. This finding is in line with a previous ADHD study involving multivariate familial factor analysis in which RTSD and MRT loaded highly on a single large factor, whereas omission and commission errors loaded highly on a separate factor (3). Strong correlations between RTSD and MRT indicate that a shared “causal” overlap may exist between the classical measures of ISV and response speed (*but* *see below*). This highlights that measures accounting for only those group differences that cannot already be explained by group differences in MRT such as “Coefficient of Variance” (CV = RTSD ÷ MRT), convey an unclear concept of variability and are thus unsuitable for ISV research in ADHD.

Despite the close relationship between RTSD and MRT or their positive associations with the ex-Gaussian ISV measures, it is noteworthy that τ shared only little variance with Gaussian μ in data of ADHD and healthy populations. These patterns point to a dissociation of slow responses (τ) from response speed or slowing (μ). In fact, in the PCA comprising of data for young healthy adults, τ and μ loaded highly on two separate components—ISV and response speed, respectively. Upon the exclusion of the ex-Gaussian measures from the PCA however, ISV and response speed measures loaded on a single factor; thus similar to a previous ADHD familial study (3). As such, it is clear that the dissociation of slow responses (τ) from slow responding (μ) disentangles ISV from response speed—highlighting the uniqueness of these RT constructs and importance of studying them separately.

The weak association found between τ with μ in both, general and clinical populations is overall in line with the patterns of patients with ADHD having elevated τ but normal or even smaller μ (**Table 1**) similar to the results reviewed by Kofler et al. (24). These results may find clinical relevance as the ex-Gaussian measures may differ in their symptomatic associations. Preliminary evidence for the same was provided by Buzy and colleagues (8) with significant associations of τ with symptoms of hyperactivity, and of μ with symptoms of both, hyperactivity and impulsivity in ADHD.

The associations of the classical ISV and response speed measures, RTSD and MRT, with the ex-Gaussian ISV measures, τ and σ, are in line with the algebraic dependency of these measures. If RTs were normally distributed, μ and σ would be equal to MRT and RTSD, respectively. However, the typical rightward skew of RT distributions, with τ modeling this ex-Gaussian portion contributes substantially to MRT as well as RTSD and thus also to its correlations with these measures. The near-perfect correlation of τ with RTSD in, both, the ADHD and healthy sample data suggest that these values may be interchangeable. This result is not surprising since the rightward skew of RT histograms, captured by τ and emerging due to the speed of a response having a physiological limit but the slowness of a response being largely uncapped, is sensitive to individual differences in variability (17).

The dissociation of σ from all other ISV measures in the PCA results for young healthy adults is striking and supports the idea that differentiated ISV measures reflect different facets of variability. In ADHD literature, elevated ISV is typically characterized by elevated τ but a few studies have also found σ to be elevated (see **Table 1**). A common explanation for elevated τ in patients with ADHD is that it reflects frequent attentional lapses due to poor suppression of the Default Mode Network [DMN; (5, 25)]. By contrast, increased σ, if given in patients with ADHD, may be an index of poor neuro-modulation or neural “noise” (26).

Finally, in the context that omission errors, similar to τ (5), may also reflect attentional lapses arising due to poor suppression of the DMN (27), it is noteworthy that, for young healthy adults, omission and commission errors formed a factor separate from those of RT-ISV and speed measures. Feige and colleagues (10) found that in children with and without ADHD, frequencies below 0.025Hz (corresponding with a cycle of 40 s) are sensitive to the quasi-periodic occurrence of particularly slow responses that contribute to τ; omission errors, however, were not sensitive to these frequencies (10). In the present work, the dimensional distinction of τ and omission errors found may be related to their different temporal dynamics and the notion that they reflect different types of attentional “lapses” (10).

A limitation of the present study is its moderate sample size for the PCA. Yet, a subject-to-item ratio above the recommended 5:1 (here, 7.8:1) (28) and the clarity of the PCA results mitigate this issue. Additionally, the study sample for the PCA mainly constituting of university students limits generalizability of findings. Replication of our findings in larger independent ADHD and control samples is thus required to conclude that slow responses, reflected by τ, can be dissociated from slow responding, as reflected by μ.

## Data Availability Statement

The raw data supporting the conclusions of this article will be made available by the authors, without undue reservation.

## Ethics Statement

The studies involving human participants were reviewed and approved by Ethics Committee, School of Psychology, Bangor University, Bangor, UK. The patients/participants provided their written informed consent to participate in this study.

## Author Contributions

GS, CS, and CK contributed conception and design of the study. GS, BF, and CS organized the database. GS performed the statistical analyses. GS wrote the first draft of the manuscript with support from CK. All authors contributed to the article and approved the submitted version.

## Funding

The article processing charge was funded by the German Research Foundation (DFG) and the University of Freiburg in the funding programme Open Access Publishing.

## Conflict of Interest

The authors declare that the research was conducted in the absence of any commercial or financial relationships that could be construed as a potential conflict of interest.

## Footnotes

1 RTSD, MRT: *r* = .94

2 τ, RTSD: *r* = .98

3 σ, RTSD: *r* = .77

4 τ, MRT: *r* = .94

5 σ, MRT: *r* = .94

6 τ, μ: *r* = −.01
